# Endotyping of non-allergic, allergic and mixed rhinitis patients using a broad panel of biomarkers in nasal secretions

**DOI:** 10.1371/journal.pone.0200366

**Published:** 2018-07-26

**Authors:** Christine L. Segboer, Wytske J. Fokkens, Ingrid Terreehorst, Cornelis M. van Drunen

**Affiliations:** Department of Otorhinolaryngology, Academic Medical Centre, Amsterdam, The Netherlands; Telethon Institute for Child Health Research, AUSTRALIA

## Abstract

**Background:**

Endotyping chronic rhinitis has proven hardest for the subgroup of non-allergic rhinitis (NAR) patients. While IgE-related inflammation is typical for allergic rhinitis (AR), no markers have been found that can be seen to positively identify NAR. A further complication is that AR and NAR might co-exist in patients with mixed rhinitis. As previous studies have considered only a limited number of inflammatory mediators, we wanted to explore whether a wider panel of mediators could help us refine the endotyping in chronic rhinitis patients.

**Objective:**

To endotype chronic rhinitis, and non-allergic rhinitis in particular, with help of molecular or cellular markers.

**Method:**

In this study we included 23 NAR patients without allergen sensitizations and with persistent rhinitis symptoms, 22 pollen sensitized rhinitis patients with seasonal symptoms, 21 mixed rhinitis patients with pollen-related symptoms and persistent symptoms outside of the pollen season, and 23 healthy controls without any symptoms. Nasal secretions were collected outside of pollen season and differences between the endotypes were assessed for a broad range of inflammatory mediators and growths factors using a multiplex ELISA.

**Results:**

Although we were able to identify two new nasal secretion makers (IL-12 and HGF) that were low in mixed and AR patients versus NAR and healthy controls, the most intriguing outcome is that despite investigating 29 general inflammatory mediators and growth factors no clear profile of non-allergic or mixed rhinitis could be found.

**Conclusion:**

Classical inflammatory markers are not able to differentiate between non-allergic or mixed rhinitis patients and healthy controls.

## Introduction

Rhinitis can be subdivided into a number of discrete pheno- and endotypes [1]. The characterization of phenotypes is hampered by limited clinical tools [2]. Identifying the molecular processes underlying rhinitis in a particular patient may help us to identify different endotypes more readily and may help to optimize treatment for these patients [3].

If we disregard infectious rhinitis, the most common rhinitis phenotype is allergic rhinitis (AR), in which a clinical response to an otherwise innocent environmental factor or allergen results in symptoms. This clinical response, in combination with specific IgE targeting aeroallergens constitutes allergic rhinitis. The second most common rhinitis phenotype is non-allergic rhinitis (NAR), which is defined as a form of non-infectious rhinitis in which it is not possible to identify an allergic component [1] [4]. NAR can be subdivided into the following phenotypes: environmental (occupational, smoking), hormones (pregnancy), medication-induced (*rhinitis medicamentosa*, NSAIDS (non-steroidal anti-inflammatory drugs), aspirin etc.), gustatory, age (rhinitis of the elderly) and/or inflammation (non-allergic rhinitis with eosinophilia syndrome (NARES) or local allergic rhinitis (LAR)). However, in a significant proportion of patients, none of these triggers are present and the disease is considered to be idiopathic [3] [1] [5]. However, the phenotypes are dynamic and overlapping, and they may evolve into one another.[1].

For a long time, nasal hyperreactivity was seen as a symptom that made it possible to differentiate between patients affected by idiopathic rhinitis (former known as *vasomotor rhinitis*) and other chronic rhinitis patients. However, we recently showed that nasal hyperreactivity is a widespread symptom that is common to both AR and NAR patients [6, 7]. Furthermore, quality of life (QoL) is equally impaired in both NAR and AR patients [8].

In addition to phenotypes, NAR can be subdivided into inflammatory, neurologic and idiopathic endotypes [4, 9] (Table 1). The prevalence of the different endotypes of NAR is unknown and this area will require research in the future. The inflammatory endotype includes two clear sub-endotypes: local allergic rhinitis (LAR) and NARES (non-allergic rhinitis with eosinophilia syndrome) [10] [1]. These two usually have a Th2 endotype involving an increase in eosinophils, IL-5, IL-4, IL-13 and, in the case of LAR, specific IgE [9] [1]. This endotype may also include the environmental phenotype (occupational, smoking)–with low-molecular-weight substances initiating a Th2 response by means of TSLP, IL-33 etc.–the drug-induced inflammatory endotype (NAIDS, aspirin) and hormonal rhinitis involving histamine H1-receptor overexpression. [1] [4] Although there is no hard evidence one can expect that the NAR inflammatory endotype will be successfully treated with a combination of intranasal corticosteroids and/or antihistamines.

**Table 1:** NAR phenotypes and endotypes.

Phenotypes [4]	Endotypes [1, 4, 9]
NARES/LAR	Th2-inflammation
Occupational	Neurogenic inflammation, Th2-inflammation
Medication-induced	Neurogenic dysbalance, Th2-inflammation, idiopathic
Hormonal	Neurogenic dysbalance, Th2-inflammation
Idiopathic rhinitis	Neurogenic inflammation, idiopathic
Rhinitis of the Elderly	Neurogenic dysbalance
Gustatory	Neurogenic dysbalance and neurogenic inflammation

In the case of the neurological endotype, one can differentiate between neurological inflammation in idiopathic rhinitis with nasal hyperreactivity and disease attributed to hyperactivity in the parasympathetic nervous system (primarily senile and gustatory rhinitis, but also, to a certain extent, hormonal and drug-induced rhinitis). In idiopathic rhinitis, studies have indicated that neurogenic signs of disease (transient potential receptor channels (TRP receptors)) affect trigeminal nerves, substance P, and calcitonin gene-related peptide [11]. Capsaicin treatment in these patients induces the reduction of TRPV-1 receptors in nasal mucosa, reducing the symptoms of nasal hyperreactivity [12] [11] [13]. Recent literature shows that azelastine (nasal anti-histamine) can achieve the same effect in TRP desensitization [14]. In patients with senile and gustatory rhinitis, ipratroprium nasal spray (atronase) reduces parasympathetic activity in the nose [15]. When all other treatments fail, vidian neurectomy may be a way of disrupting the parasympathetic innervation of the nose and stopping rhinorrhea [16].

We are not aware of a type 1 or type 3 inflammatory endotype (INF-y, IL-17, TNF) in NAR; type 1 inflammation would seem to be related mainly to infectious rhinitis and not to NAR phenotypes [4].

It is also important to realize that the underlying mechanism of NAR can also be present in AR. When there is a seasonal allergen sensitization accompanied by perennial symptoms, symptoms outside the pollen season may be the result of ongoing, minimal persistent, allergic inflammation or the same underlying mechanism as in NAR may be responsible for symptoms [9, 17].

Studies to assess single cellular or molecular markers (or combinations of these markers) with the aim of defining the endotype of AR and NAR are scarce [3] [18]. Unfortunately, it is difficult to combine the data from these studies due to differences in the inclusion criteria that resulted in unclear phenotypes. In general terms, these studies have mainly identified differences between AR and NAR that are linked to cells and markers related to IgE inflammation in AR: total and specific IgE, eosinophils, mast cells, and IL-5 [19]. However, some of these studies showed comparable levels of allergic inflammation in AR and NAR patients, possibly indicating some form of local allergic inflammation in these NAR patients [20] [21]. In cases where idiopathic rhinitis patient were studied, levels of inflammatory mediators or cells were found to be the same as in healthy controls [22].

We wondered whether the endotyping of chronic rhinitis patients–and non-allergic rhinitis patients in particular–with molecular or cellular markers could be helpful. In this cross-sectional study we studied nasal secretions for the presence of potentially relevant mediators related to different rhinitis endotypes. We looked at non-allergic rhinitis patients (selected to represent idiopathic rhinitis), grass-pollen-allergic rhinitis patients (outside allergen exposure) as an example of minimal persistent, allergic inflammation, mixed rhinitis patients and healthy controls [17].

## Material and methods

### Inclusion and exclusion criteria

Patients were recruited from the outpatient clinic of the Department of Otorhinolaryngology of the Academic Medical Center, Amsterdam, the Netherlands. Medical ethical approval was obtained (MEC 08/356) by the Institutional Medical Ethics Review Committee (MRTC) of the Academic Medical Centre of Amsterdam (AMC) and all participants gave their written informed consent. A patient information document (approved by the Institutional Medical Ethics Review Committee) was signed per included patient. All rhinitis patients had a positive history of rhinitis symptoms at least one year and were referred to our tertiary care outpatient clinic by their general practitioner or another otorhinolaryngology clinic.

Only pollen-sensitized AR patients were included, excluding AR patients with a perennial allergen sensitization like for example house dust mite. Pollen-sensitized AR patients had at least one positive SPT result for a pollen allergen (defined as: a wheal equal in size or larger than 3 mm and no response to the negative control) and clinical symptoms relevant to their sensitization and no symptoms outside the pollen season when they were included in the study. NAR was defined as clinically relevant symptoms of rhinitis without a positive SPT. In this study, we selected non-allergic rhinitis patients and excluded smoking, senile, gustatory, occupational, medication-induced and pregnancy rhinitis. Mixed rhinitis patients had perennial rhinitis symptoms with peak symptoms during the pollen season and a positive SPT for one or more pollen allergens, mixed rhinitis patients with perennial allergen sensitizations were excluded as well. The healthy control group had no symptoms of rhinitis and a negative SPT.

The exclusion criteria for all patient groups were anatomic abnormalities, or any systemic disease or medication influencing nasal function. Patients had to be free of symptoms of upper airway infection for at least 1 week. Patients with symptoms of chronic rhinosinusitis (CRS) (diagnosed according to EPOS criteria, i.e. two or more symptoms of the following: nasal congestion/blockage, (anterior/posterior) rhinorrhea, hyposmia/anosmia, facial pain/pressure; with at least either nasal congestion or rhinorrhea, combined with signs of CRS with nasal endoscopy and/or CT sinus) with or without nasal polyposis were excluded, as were patients who had undergone nasal surgery in the previous 3 months, patients with a history of immunotherapy or asthma, and patients who smoked [23].

### Study design

#### Data collection

Nasal secretions were collected to compare molecular biological parameters in nasal secretions of 23 pollen-sensitized AR patients outside the season, 23 symptomatic NAR patients, 23 symptomatic pollen-sensitized mixed rhinitis patients and 23 healthy controls. Nasal secretions were obtained outside the pollen season from September to March-May, with the latter limit depending on the patient’s seasonal sensitizations.

#### Screening visit

Patients were seen for a screening visit and a sampling visit, both outside the pollen season. Patients were asked to stop with their antihistamines for 48 hours before both visits and with nasal corticosteroid medication or any other medication influencing nasal function for at least 4 weeks. The screening visit included a skin prick test (SPT), and an Ear-Nose-Throat (ENT) history and examination. Patients were categorized using the ARIA classification system [24].

#### Sampling visit

The sampling visit included an assessment of nasal symptoms (rhinorrhea, nasal congestion, itch and sneezing) with a Visual Analogue Scale (VAS) (maximum 100 mm per nasal symptom) and a nasal airflow assessment based on Peak Nasal Inspiratory Flow (PNIF). To collect the secretion, a small merocel (Ivalon, ThinPack™) was inserted into the inferior meatus of one nostril for three minutes. Which nostril was used depended on the anatomical situation of the individual patient. The merocel was weighed before and after application to calculate total secretion weights and the secretion was eluted by soaking in 3 mL (0.9% w/v) NaCl at 4°C for two hours and collected after centrifugation for 15 min at 1,500g. Aliquots were stored at -80°C until use in a multiplex ELISA.

#### Protein multiplex ELISA

The samples collected from the three patient groups and healthy control group were used to determine protein levels for a broad range of inflammatory mediators and growth factors (the lower detection limit for each mediator is stated in pg/mL between brackets): IL-1RA (13.4 pg/mL), IL-1β (3.7 pg/mL), IL-2R (10.5 pg/mL), IL-2 (4.7 pg/mL), IL-4 (16.6 pg/mL), IL-5 (4.2 pg/mL), IL-6 (2.7 pg/ml), IL-8 (2.4 pg/mL), IL-10 (9.5), IL-12 (4.6), IL-13 (5.0), IL-15 (11.0), IL-17 (8.6 pg/mL), eotaxin (1.6 pg/mL), TNF-α (3.9 pg/mL), INF-α (6.7 pg/mL), IFN-γ (11.0 pg/mL), MCP-1 (4.9) pg/mL), GM-CSF (10.3 pg/mL), G-SCF (26.8 pg/mL), VEGF (3.5 pg/mL), FGF-β (1.6 pg/mL), EGF (3.3 pg/mL), HGF (6.8 pg/mL), IP-10 (1.9 pg/mL), MIG (1.7 pg/mL), RANTES (5.4 pg/mL), MIP1-α (6.9 pg/mL), MIP1-β (3.3 pg/mL).

**Fig 1:**
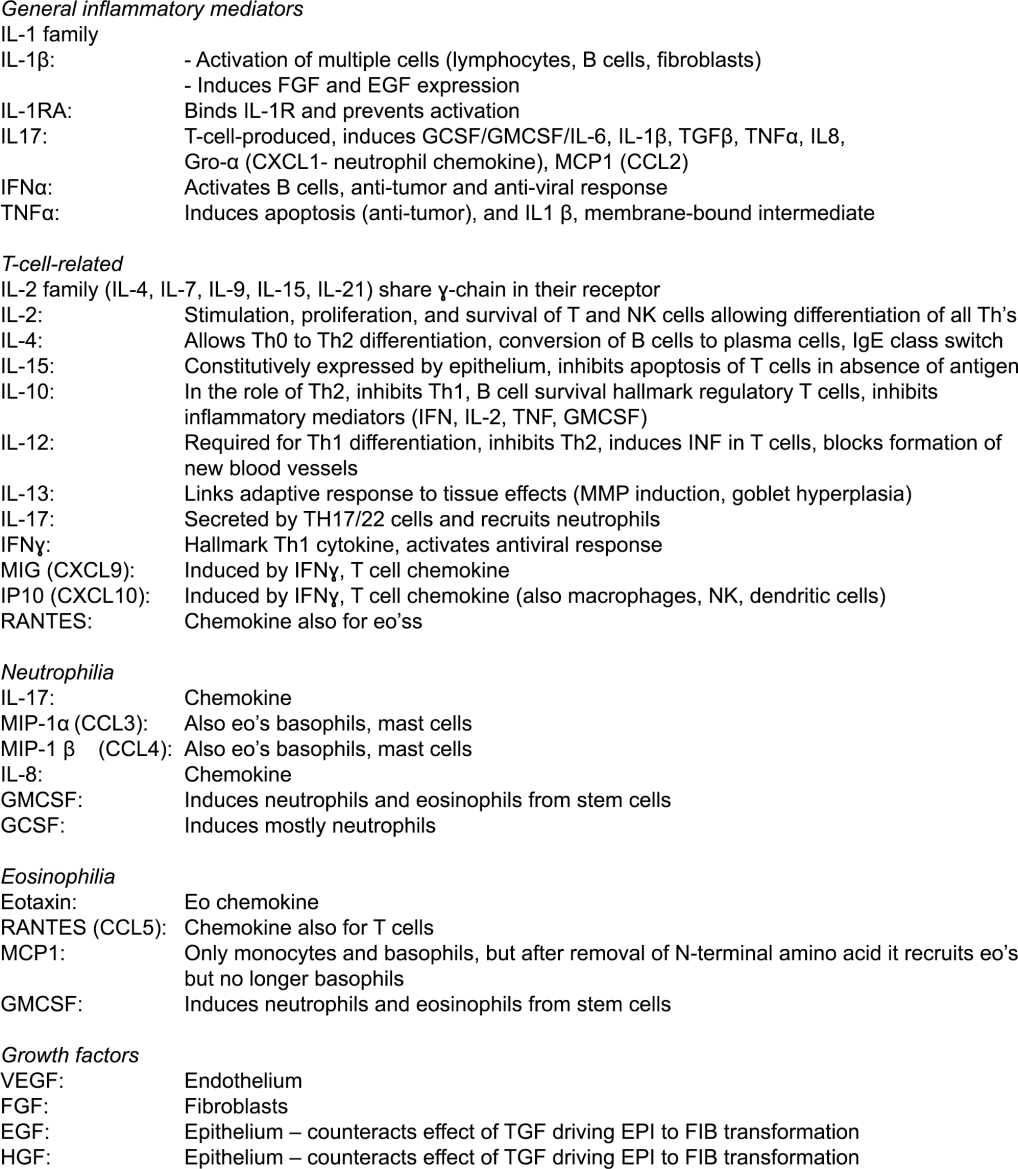
Function of measured cytokines.

The function of the measured cytokines and their relation to an underlying endotype can be found in Fig 1.

Cytokine levels were measured with a Human Cytokine Thirty-Plex Antibody Bead Kit (Biosource, USA) in combination with a Bio-Plex workstation (Bio-Rad, NL). All standards were diluted in HBSS medium as required by the manufacturer. All standards were diluted in HBSS medium (3 mL) as required by the manufacturer. Luminex software was employed for the protein concentration calculations and all these concentrations–after correcting for the different amounts of nasal secretions collected- were expressed in pg/mL.

#### Statistics and principal component analysis

SPSS 20.0 (Chicago, IL, USA) was used for statistical analysis. Cytokine, chemokine, and growth factor values that were below the detection limits were recoded to the lowest measurable value. The distribution of the data was not normal and Kruskal-Wallis non-parametric tests were therefore performed to check for significant between-group variability. Where significant between-group variability was found, Mann-Whitney-U non-parametric tests were performed for between-group comparisons. The level of statistical significance was set to <0.003 after Bonferroni correction for multiple testing (0.05/15). Principal component analysis involving the extraction of 11 components in a rotated component matrix (Varimax with Kaiser normalization) took place to determine whether a combination of cytokines could distinguish between groups of patients.

## Results

### Characterization of participants

The patient groups were comparable in terms of age, gender and percentage of patients with moderate-severe persistent disease (Table 2).

**Table 2:** Patient characteristics.

	Healthy Controls (n = 23)	Mixed Rhinitis (n = 21)	Non-Allergic rhinitis (n = 23)	Allergic rhinitis (outside season) (n = 22)	Significance levels over the groups
Gender (male %)	34.8%	19.0%	34.8%	36.4%	*p* = *0*.*540* ^a^
Mean age (years)	32.7	38.8	41.7	36.2	*p* = *0*.*160* ^b^
ARIA class. (% moderate-severe persistent)	Not relevant	85.7%	82.6%	68.2%	*p* = *0*.*230* ^a^
Allergen sensitization	n.a.	Only trees (23.8%)	n.a.	Only trees (9.1%)	
		Only grass (23.8%)		Only grass (31.8%)	
		Only plants (4.8%)		Only plants (0%)	
		Mixture of above: (47.6%)		Mixture of above: (59.1%)	
VAS rhinorrhea					*p = 0*.*185* *†*
Median (range)	0.0 (0–28)	0.0 (0–79)	2.0 (0–88)	0.0 (0–48)	
Mean (SD)	5.4 (8.5)	12.1 (25.0)	13.0 (23.9)	4.7 (11.5)	
VAS congestion				|	*p = 0*.*001†*
Median (range)	1.0 (0–51)	14.0 (0–83)	30.0 (0–98)	3.5 (0–47)	
Mean (SD)	6.4 (11.7)	21.1 (25.1)	41.2 (38.1)	9.0 (12.5)	
VAS itch					*p = 0*.*011†*
Median (range)	0.0 (0–23)	2.0 (0–80)	1.0 (0–67)	0.0 (0–29)	
Mean (SD)	2.5 (5.5)	17.1 (24.7)	11.7 (20.3)	3.0 (7.4)	
VAS burning					*p = 0*.*012†*
Median (range)	0.0 (0–2.0)	0.0 (0–63)	0.0 (0–71)	0.0 (0–7)	
Mean (SD)	0.26 (0.54)	10.5 (19.9)	7.4 (16.9)	0.4 (1.5)	
VAS sneezing					*p = 0*.*160†*
Median (range)	0.0 (0–33)	0.0 (0–69)	1.0 (0–60)	0.0 (0–40)	
Mean (SD)	2.7 (7.4)	8,4 (18.8)	9.6 (17.4)	4.7 (10.3)	
PNIF					*p = 0*.*052†* *p = 0*.*446* *‡*
Median (range)	140.0 (70–260)	105.0 (40–230)	110.0 (60–270)	115.0 (40–200)	
Mean (SD)	147.4 (45.8)	107.9 (50.7)	125.9 (48.0)	120.7 (41.4)	

a: Chi Square Test

b: Kruskal Wallis

*†*: Kruskal Wallis over all groups

*‡*: Kruskal Wallis over patient groups only

The NAR and the mixed rhinitis group had significantly (p <0.001) higher total VAS scores and VAS scores for congestion and itch (*p*< 0.001 and *p*< 0.011 respectively) than healthy controls and allergic rhinitis patients (outside the season). Although the PNIF was higher in the healthy controls than in the patient groups, the differences were not statistically significant. Two patients in the mixed rhinitis group and one in the allergic rhinitis group were excluded: one patient in the mixed rhinitis group started smoking again and the other was found to have a perennial allergen sensitization for cat combined with daily exposure to this allergen, and the bothersome symptoms of rhinitis disappeared in the patient in the AR group.

### Nasal secretion analysis

#### Nasal secretions of non-allergic rhinitis patients cannot be differentiated from healthy controls

Above the detection levels were: IL-8, IL-12, IL1RA, MCP1, MIP1α, MIP1β, EGF, VEGF, HGF, INFα, RANTES, IP10, IL7, FGF- β and MIG. No significant differences were found between non-allergic rhinitis and healthy controls for any of the mediators above detection level. The functions of these mediators can be found in Fig 1.

#### Levels of IL-12 and HGF are low in nasal secretions of mixed and allergic rhinitis patients

**Fig 2:**
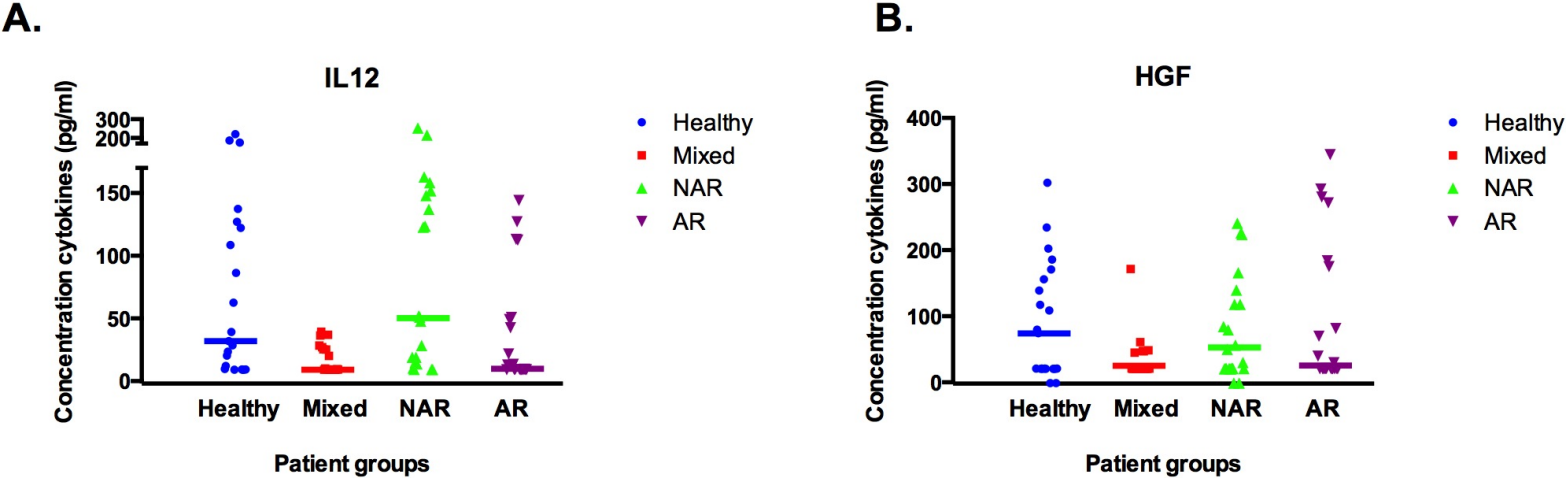
**(A and B). Cytokines that were (significantly) different between groups: IL12 (A) and HGF (B).** (A and B): Expression of cytokines (pg/mL) in nasal lavage fluid in a healthy control group and in mixed, non-allergic (NAR) and allergic (AR) rhinitis patient groups. Individual concentrations are represented with a symbol; median concentration levels per group are represented with a horizontal line. * IL12 is significantly lower in mixed versus healthy controls. ** IL12 is significantly lower in AR and mixed versus NAR.

There was a significant difference between patient groups (Kruskal-Wallis, *p* < 0.003) for two mediators above detection level, IL-12 and HGF (Table 3). Median IL-12 levels were significantly higher in the NAR group than in the AR and mixed groups. Median IL-12 levels in AR and mixed rhinitis were lower than in healthy controls but they reached significance in the mixed patient group only. A similar pattern was seen for HGF but it was not found to be significant when we set the level of significance at *p* < 0.003 for the correction of multiple testing (Fig 2A and 2B).

**Table 3:** Cytokine levels in nasal secretions.

Mediator	Controls Median (*range*) Mean *(SD)*	Mixed Rhinitis Median (*range*) Mean *(SD)*	Non-Allergic Median (*range*) Mean *(SD)*	Allergic Median (*range*) Mean *(SD)*	Kruskal Wallis (*p)*
EGF	8.0 *(3*.*4–12*.*5)* 9.2 *(4*.*8)*	3.5 *(3*.*4–67*.*5)* 13.6 *(15*.*2)*	5.1 *(3*.*4–20*.*2)* 6.2 *(3*.*9)*	3.4 *(3*.*4–37*.*8)* 10.4 *(9*.*7)*	*p =* 0.392
HGF	74.6 *(74*.*6–301*.*9)* 110.2 *(84*.*0)*	20.6 *(20*.*5–171*.*4)* 41.1 *(32*.*1)*	50.1 *(20*.*8–240*.*5)* 88.2 *(71*.*5)*	20.8 (*20*.*5–344*.*6)* 96.2 *(107*.*4)*	*p =* 0.021
IL-12	32.0 *(20*.*3–221*.*1)*^*a*^ 69.4 *(67*.*8)*	9.30 (*9*.*2–39*.*4)*^*a*^ ^,^^*b*^ 19.4 *(9*.*5)*	51.1 *(9*.*3–253*.*0)*^*b*^ 84.1 (76.7)	10.8 *(9*.*2–144*.*2)*^*b*^ 38.1 *(43*.*6)*	*p =* 0.001
IL-8	357.1 *(567*.*9–3667*.*1)* 835.5 *(1032*,*5)*	217.2 *(250*.*0–2072*.*5)* 441.5 *(559*.*7)*	254.7 *(159*.*0–1681*.*3)* 452.4 *(491*.*5)*	509.9 *(51*.*7–3072*.*6)* 785.3 (849.8)	*p =* 0.148
RANTES	21.7 *(16*.*2–41*.*3)* 22.2 *(13*.*0)*	16.4 *(16*.*2–57*.*7)* 22.5 *(12*.*6)*	16.7 *(16*.*3–27*.*3)* 17.7 *(6*.*0)*	16.4 *(16*.*2–44*.*7)* 18.9 *(6*.*4)*	*p =* 0.562
IL-1RA	311.8 *(39*.*8–1583*.*5)* 360.9 *(338*.*1)*	151.6 *(39*.*9–2431*.*9)* 355.3 *(545*.*0)*	194.0 *(39*.*9–2587*.*7*) 335.9 *(542*.*1)*	179.4 *(39*.*9–1111*.*9)* 334.0 *(314*.*4)*	*p =* 0.622
INF-α	42.6 *(6*.*2–77*.*2)* 38.7 *(24*.*1)*	6.3 *(6*.*2–79*.*5)* 28.6 *(23*.*1)*	43.9 *(6*.*2–81*.*6)* 40.9 *(26*.*3)*	6.3 *(6*.*2–99*.*9)* 33.5 *(27*.*3)*	*p =* 0.108
MCP1	38.1 *(14*.*9–128*.*3)* 46.2 *(31*.*5)*	33.7 *(14*.*8–186*.*0)* 61.7 *(58*.*0)*	24.3 (*5*.*0–120*.*3)* 30.5 *(24*.*1)*	32.6 *(14*.*9–134*.*7)* 47.7 *(38*.*2)*	*p =* 0.336
IP10	45.0 *(6*.*3–863*.*0)* 131.8 *(199*.*3)*	20.5 *(5*.*6–2447*.*5)* 212.9 *(533*.*0)*	28.8 *(5*.*7–1465*.*1)* 69.1 *(105*.*9)*	39.0 *(5*.*7–1195*.*1)* 124.4 *(273*.*1)*	*p =* 0.336
MIG	22.4 *(5*.*1–77*.*5)* 131.8 *(199*.*3)*	16.3 *(5*.*1–218*.*1)* 212.9 *(533*.*0)*	22.5 (*5*.*1–65*.*5)* 69.1 *(105*.*9)*	17.3 *(5*.*1–106*.*0)* 124.4 *(273*.*1)*	*p =* 0.720
MIP-1β	10.0 *(9*.*9–22*.*1)* *12*.*1 (3*.*7)*	9.9 *(9*.*9–13*.*4)* 10.8 *(0*.*9)*	10.0 *(9*.*9–18*.*4)* 11.1 *(2*.*2)*	10.0 *(9*.*9–20*.*5)* 11.7 *(3*.*0)*	*p =* 0.358
MIP-1α	20.8 *(20*.*6–41*.*9)* 25.9 *(6*.*6)*	20.7 *(20*.*6–22*.*8)* 23.0 *(2*.*3)*	20.8 *(20*.*6–50*.*2)* 26.1 *(8*.*0)*	20.8 *(20*.*6–47*.*7)* 25.9 *(6*.*7)*	*p =* 0.091
VEGF	5.1 *(3*.*4–24*.*0)* 8.8 *(4*.*9)*	3.4 *(3*.*4–12*.*3)* 8.1 *(3*.*4)*	4.3 *(3*.*4–10*.*1)* 5.7 *(2*.*5)*	3.4 *(3*.*4–37*.*9)* 9.0 *(8*.*3)*	*p =* 0.065
IL-15	33.2 *(32*.*9–276*.*0)* 57.7 *(56*.*1)*	33.4 *(32*.*9–418*.*0)* 84.8 *(91*.*7)*	33.1 *(32*.*9–68*.*6)* 37.1 *(10*.*1)*	33.2 *(32*.*9–417*.*4)* 66.7 *(90*.*3)*	*p =* 0.568
IL-7	9.8 *(9*.*7–157*.*9)* 58.3 *(46*.*7)*	9.8 *(9*.*7–177*.*2)* 59.8 *(52*.*7)*	9.8 *(9*.*7–67*.*3)* 56.7 *(49*.*4)*	10.1 *(9*.*7–174*.*4)* 52.9 *(50*.*7)*	*p =* 0.583
FGF-β	4.8 (*4*.*7–12*.*9*) 11.2 (2.8)	4.8 *(4*.*7–9*.*6)* 9.6 *(3*.*4)*	4.8 *(4*.*7–5*.*1)* 11.8 *(2*.*3)*	4.8 *(4*.*8–9*.*6)* 10.1 *(3*.*5)*	*p =* 0.782

*a*: Kruskal Wallis *p* < 0.003 mixed versus healthy

*b*: Kruskal Wallis *p* < 0.003 AR and mixed versus NAR

#### Principal component analysis reveals high inter- and intra-group variance as a consequence of the large dynamic range of expression for multiple mediators

As has been shown above, some of the individual cytokines showed significant differences between the groups, but neither the three patient groups nor the healthy controls expressed a feature cytokine. We used principal component analysis to explore whether the use of combinations of mediators rather than single mediators could improve molecular characterization for our study population.

**Fig 3:**
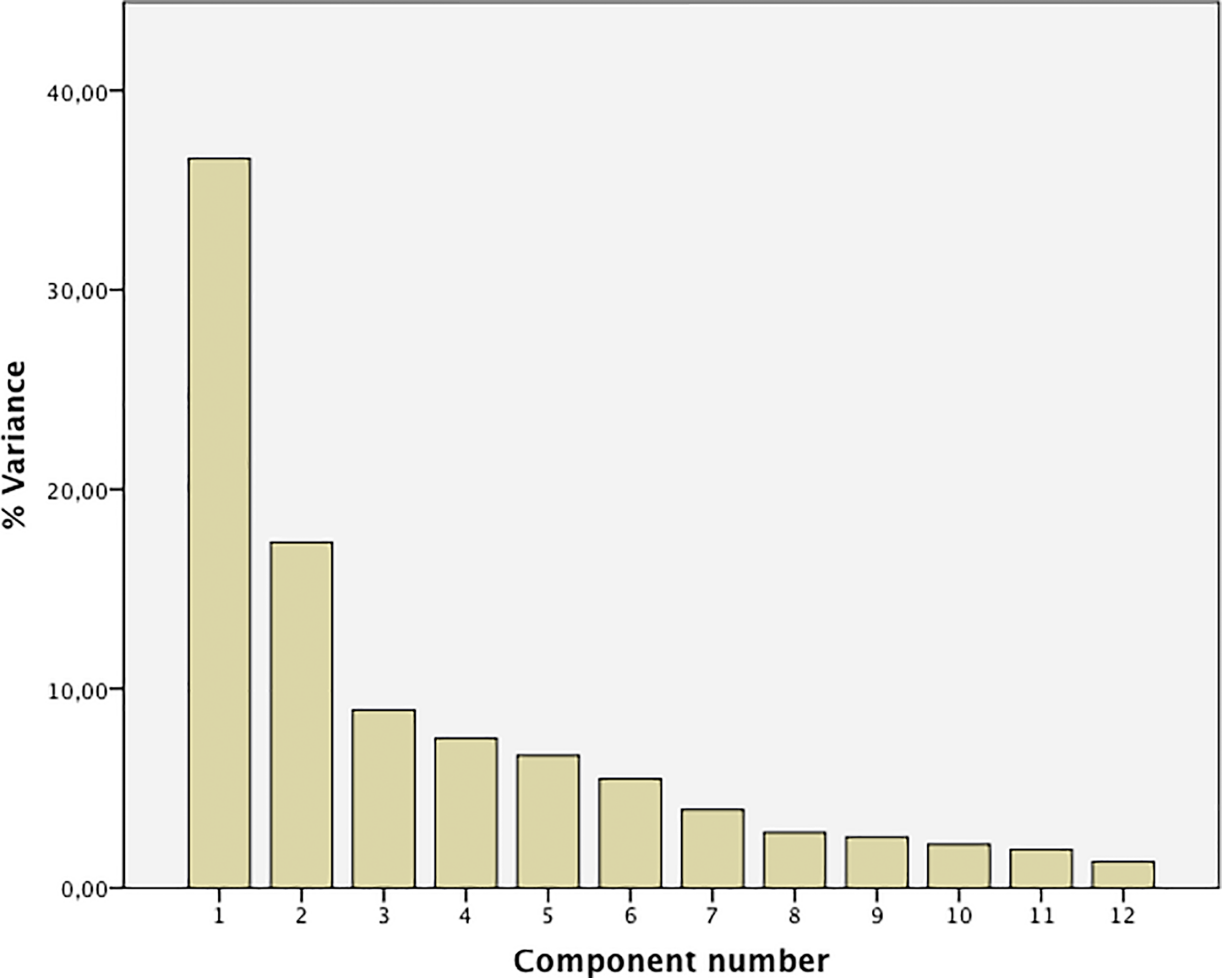
Proportion of contribution to total variance of principal components.

Mapping individual patients in multi-dimensional space showed that we could reduce the dataset to the first five principal components with eigenvalues over 1 that together account for more than 75% of total variance (Fig 3) in all samples.

**Fig 4:**
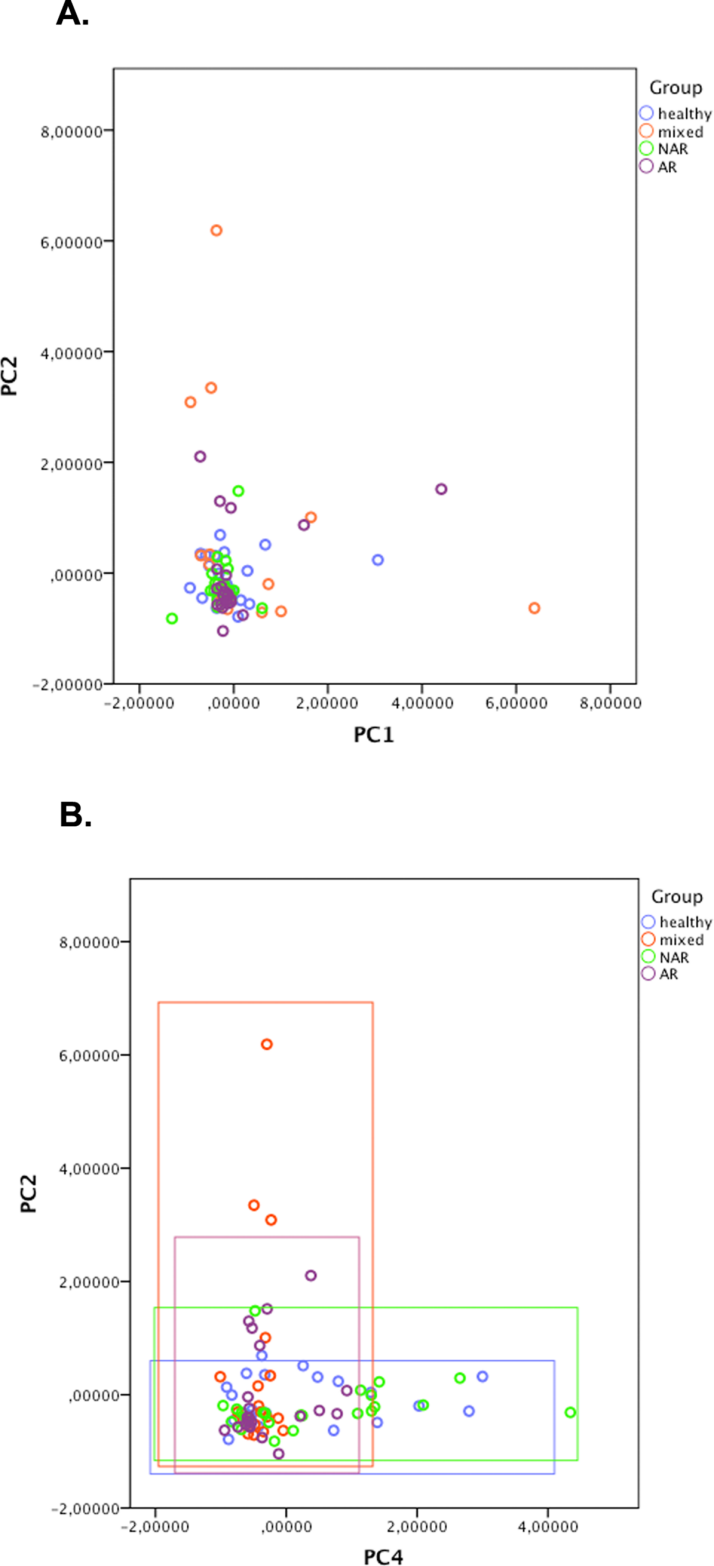
**(A and B). Principal component analysis.** (A): PC1 versus PC2: no distinction between groups (B): PC2 versus PC4: distinction between mixed and AR patients (with low values for PC4) and healthy and NAR (with low values for PC2).

Table 4 shows the relative contribution of each of the mediators to these five principal components. The first three principal components showed significant loading of more than 0.5 for multiple mediators: MCP1, IP10, and IL-15 on PCA1, EGF and MCP1 on PCA2, and the related mediators MIP1α and MIP1β on PCA3. The last two principal components seemed to depend on single mediators: PCA4 on IL-12 and PCA5 on IL-17. Pairwise plotting of the five principal components did not result in a full separation of an individual patient group; the plot of PCA1/PCA2 is shown in Fig 4A as an example. The best separation of groups was obtained when PCA4 was involved. For instance, plotting PCA2 against PCA4 (Fig 4B) revealed the unique low vales for the mixed rhinitis group.

**Table 4:** Contribution of individual cytokines to the principal components.

Cytokine	PCA 1	PCA 2	PCA 3	PCA 4	PCA 5
EGF	+ 0.15	**+ 0.94**	+ 0.07	- 0.01	+ 0.05
HGF	+ 0.04	+ 0.09	+ 0.34	+ 0.38	+ 0.22
IL12	- 0.04	- 0.02	+ 0.19	**+ 0.95**	- 0.03
IL8	0	+ 0.18	+ 0.11	+ 0.10	+ 0.04
RANTES	+ 0.29	+ 0.06	+ 0.22	+ 0.11	+ 0.23
IL1RA	+ 0.32	+ 0.13	+ 0.04	- 0.03	+ 0.03
INFA	+ 0.06	- 0.05	+ 0.22	+ 0.03	+ 0.21
MCP1	**+ 0.53**	**+ 0.65**	+ 0.10	- 0.02	+ 0.09
IP10	**+ 0.94**	+ 0.10	+ 0.01	0	- 0.02
MIG	+ 0.33	+ 0.25	+ 0.02	+ 0.04	+ 0.14
MIP1B	+ 0.05	0.09	**+ 0.91**	+ 0.19	+ 0.02
MIP1A	+ 0.05	0.05	**+ 0.58**	+ 0.04	+ 0.09
VEGF	+ 0.12	0.23	+ 0.21	+ 0.17	+ 0.19
IL15	**+ 0.92**	0.02	+ 0.08	- 0.03	+ 0.12
IL7	+ 0.08	0.07	+ 0.03	- 0.01	**+ 0.96**
FGFB	0.07	0.17	+ 0.27	- 0.01	+ 0.07

We evaluated potential correlations between mediators on the basis of the significant loadings of multiple mediators for each principal component. Indeed, Pearson’s correlation coefficient was highly significant for mediators that weighted PCA1 (MCP1, IP10, and IL-15): MCP1 versus IP10 (r = 0.642, *p* = < 0.0001), MCP1 versus IL-15 (r = 0.669, *p* = < 0.0001), and IP10 versus IL-15 (r = 0.907, *p* = < 0.0001). The same picture emerges for the mediators weighting PCA2 (EGF versus MCP1, r = 0.776, *p* = < 0.0001) and for MIP1α versus MIP1β (r = 0.764, *p* = < 0.0001) on PCA3.

## Discussion

The most important outcome of this study is that, looking at a wide panel of mediators related to endotypes of (general/Th2/Th1) inflammation, no clear profile could be found in non-allergic rhinitis patients. In the past, only a limited panel of mainly Th2 mediators–examples being IL-5, IgE, and eosinophils–were taken into account when comparing non-allergic rhinitis patients with allergic rhinitis patients [18]. The aim of this study was to broaden that panel in order to establish a wider picture, and to assess a large number of mediators and chemokines related to growth, inflammation (general/Th1/Th2/Th3), eosinophils, and neutrophils. In NAR patients, none of the levels of these mediators differed significantly from those in healthy controls, emphasizing that no distinction can be made between the inflammatory patterns in non-allergic rhinitis (mainly idiopathic rhinitis) and healthy controls (Table 3). We have to acknowledge that in this study we have not looked at the typical neurogenic inflammatory markers in NAR patients. This might have shown a distinctive pattern in NAR compared to healthy controls.

On a phenotype level, it is important to realize that -although we excluded NAR patients with senile, gustatory, occupational, medication-induced and pregnancy rhinitis- we cannot objectively claim to have excluded NARES or LAR. The history of NAR patients was however not suggestive for an allergen sensitization. Differentiating at a phenotype level between AR–particularly when there is concomitant perennial sensitization–and mixed rhinitis can be extremely complicated. AR and mixed patients in this study were therefore pollen-sensitized only and the study was performed outside the pollen season. At the phenotype level, this meant that AR patients did not have symptoms during the study but that mixed and NAR patients did and that molecular differences between AR, NAR and mixed patients were independent of allergic inflammation. We aimed to identify, where present, the specific endotype of the NAR profile in NAR and mixed patients.

In a broad panel of inflammatory mediators, the mediator profile of non-allergic rhinitis patients resembles that of healthy controls whereas the profile of mixed rhinitis patients resembles that of allergic rhinitis patients. The analysis of multiple inflammatory mediators did reveal differences in the levels of the mediators IL-12 and HGF (which were significantly lower in AR and mixed rhinitis than in NAR and healthy controls) that have not been extensively explored previously in the context of different forms of rhinitis.

Traditionally, IL-12 has been seen as a hallmark Th1 cytokine produced by dendritic cells that skews native T lymphocytes to produce INF-gamma. As we know, there is cross-regulation of anti-viral Th1 and allergic Th2 responses by the mutual inhibitory effects of IL-12 on Th2, and IL-4 on Th1, responses. It might therefore be assumed that, in our study, the AR patients with a predominant Th2-skewed inflammation would have lower levels of IL-12 than healthy controls or non-allergic rhinitis patients. Nevertheless, IL-12 levels in these pollen-allergic patients were very low, even when taking into consideration the fact that these patients were seen outside of the pollen season. We also failed to detect IL-4, IL-5, and IL-13 in nasal secretions, which suggest that it is unlikely that low IL-12 levels are a result of active Th2-dominated inflammation. This suggests that the low IL-12 levels could be an intrinsic characteristic of pollen-allergic patients.

A similar pattern in the nasal secretion levels was seen for HGF. This mediator is known to regulate dendritic cell migration, inhibit epithelial apoptosis, and reduce airway eosinophilia in OVA-allergic mice [25] [26]. HGF can also suppress IL-13-induced eotaxin expression in airway epithelial cells [27] [28]. The low level of HGF may therefore facilitate Th2 responses in AR and mixed rhinitis patients.

The overall low expression of IL-12 and HGF in mixed rhinitis patients may be a small step towards this goal and it does show that a more unbiased approach may help to reveal new aspects of a disease that have not been previously considered.

Although IL-12 is best known in relationship to dendritic cells, it has been shown that other cells such as epithelium produce IL-12 in substantial quantities; we have shown that IL-12 expression in an epithelial cell line can be up-regulated through the activation of the cells by pollen allergen [29] [30]. Epithelial cells can also secrete HGF, which is in line with the concept that proteins from the nasal epithelium could dominate the protein content of nasal secretion. The potential contribution of nasal epithelium and the potential intrinsically low level of IL-12 and HGF in pollen-allergic individuals outside the pollen season could concur with our observations of the nasal epithelium of HDM-allergic individuals, which seems to stay activated when these cells are cultured *ex vivo* in the absence of allergen exposure [31]. How the differences we have observed may contribute to the pathological mechanisms of allergic rhinitis or idiopathic rhinitis remains to be explored. However, low levels of IL-12 and HGF could facilitate a stronger Th2 response upon allergen exposure. In addition to the obvious targets of the nasal mediators we may also need to consider potential contributions from the family of innate lymphoid cells. Type 2 innate lymphoid cells (ILC2) play an important role in chronic inflammatory airway diseases (such as chronic rhinosinusitis and diseases of the lower airways). IL-12 and IL-4 can switch the function of ILC2 into either type 1 or type 2 inflammation [32], [33].

Also looking at groups of mediators instead of only individual mediators–as was done in the principal component analysis- did not help us in differentiation between patient groups. Principal component analysis showed us that the cytokines present on the first principal component contribute most to variation between patient groups. The clinical implications, however, remain unknown as the numbers and types of cytokines and/or the defined sets of phenotypes cannot differentiate between groups of patients.

In conclusion, looking at a broad panel of mediators did not allow us to identify a mediator profile that links non-allergic rhinitis to a general or Th2/Th1 inflammatory or neurogenic endotype. Nor could we identify a specific combination of mediators that differentiate between non-allergic rhinitis and healthy controls. This confirms previous data that looked at a limited number of mediators in idiopathic rhinitis patients (mainly at IgE, eosinophils, IgE, mast cells) and found no differences from healthy controls [19, 22].

The panel of inflammatory mediators was still limited in number and function: we did not look at, for example, neuropeptides such as SP or CGRP as markers of neurogenic inflammation. Further research to assess differences in a completely unbiased way–in other words at the mRNA level (micro-array) and including mediators related to neurogenic inflammation–may help us to identify the distinct features of this patient group, for which treatment is unsatisfactory owing to our lack of understanding of the underlying etiology.

## Supporting information

S1 File (XLS)
